# 3D vs 2D laparoscopic radical prostatectomy in organ-confined prostate cancer: comparison of operative data and pentafecta rates: a single cohort study

**DOI:** 10.1186/s12894-015-0006-9

**Published:** 2015-02-21

**Authors:** Pierluigi Bove, Valerio Iacovelli, Francesco Celestino, Francesco De Carlo, Giuseppe Vespasiani, Enrico Finazzi Agrò

**Affiliations:** Department of Urology, Tor Vergata University of Rome, V.le Oxford 81, 00133 Rome, Italy

**Keywords:** Laparoscopic radical prostatectomy, Pentafecta, 3D laparoscopy, Prostatectomy

## Abstract

**Background:**

Currently, men are younger at the time of diagnosis of prostate cancer and more interested in less invasive surgical approaches (traditional laparoscopy, 3D-laparoscopy, robotics). Outcomes of continence, erectile function, cancer cure, positive surgical margins and complication are well collected in the pentafecta rate. However, no comparative studies between 4th generation 3D-HD vision system laparoscopy and standard bi-dimensional laparoscopy have been reported. This study aimed to compare the operative, perioperative data and pentafecta rates between 2D and 3D laparoscopic radical prostatectomy (LRP) and to identify the actual role of 3D LRP in urology.

**Methods:**

From October 2012 to July 2013, 86 patients with clinically localized prostate cancer [PCa: age ≤ 70 years, prostate-specific antigen (PSA) ≤ 10 ng/ml, biopsy Gleason score ≤ 7] underwent laparoscopic extraperitoneal radical prostatectomy (LERP) and were followed for approximately 14 months (range 12–25). Patients were selected for inclusion via hospital record data, and divided into two groups. Their patient records were then analyzed. Patients were randomized into two groups: the former 2D-LERP (43 pts) operated with the use of 2D-HD camera; the latter 3D-LERP (43 pts) operated with the use of a 3D-HD 4th generation view system. The operative and perioperative data and the pentafecta rates between 2D-LERP and 3D-LERP were compared.

**Results:**

The overall pentafecta rates at 3 months were 47.4% and 49.6% in the 2D- and 3D-LERP group respectively. The pentafecta rate at 12 months was 62.7% and 67% for each group respectively. 4th generation 3D-HD vision system provides advantages over standard bi-dimensional view with regard to intraoperative steps. Our data suggest a trend of improvement in intraoperative blood loss and postoperative recovery of continence with the respect of the oncological safety.

**Conclusions:**

Use of the 3D technology by a single surgeon significantly enhances the possibility of achieving better intraoperative results and pentafecta in all patients undergoing LERP. Potency was the most difficult outcome to reach after surgery, and it was the main factor leading to pentafecta failure. Nevertheless, further studies are necessary to better comprehend the role of 3D-LERP in modern urology.

## Background

Prostate cancer is the most common tumor in people aged over 50 and is the second leading cause of cancer death in Europe and in the United States. Worldwide, nearly 900,000 men were estimated to have been diagnosed with prostate cancer during 2008 and 258,000 men died for this reason [1]. Incidence in Western countries is higher than in less developed ones where it is slowly increasing. Furthermore, there were recent significant decreases in prostate cancer mortality in Europe and in the United States. In contrast, mortality rates have increased in other countries [2,3]. Decreasing mortality rates is mainly due to earlier diagnosis and improved treatment.

Laparoscopic radical prostatectomy (LRP) has become an established treatment for organ confined prostate cancer and is increasingly performed at selected centers worldwide even though open radical retropubic prostatectomy (RRP) is widely considered the treatment of choice. For the first time in 1992 Schuessler carried out a LRP in order to transfer the well-known advantages of the laparoscopic technique to the most common open surgical treatment for prostate cancer [4]. Only after years, Guillonneau and Valencien improved the techniques obtaining results similar to those of open surgery but, because of the steep learning curve, laparoscopic radical prostatectomy has slowly risen in popularity [5]. The advent of robotic surgery has further helped to confine laparoscopic surgery to a special niche. Shorter learning curve, three-dimensional view as well as the ease of movement offered by the Da Vinci® operating arms have made robot-assisted laparoscopic prostatectomy (RALP) more reproducible despite the higher costs. So RALP is easier to learn and is now the surgical treatment of choice in most centers of excellence in the United States [6].

Nowadays, laparoscopic surgery could be regenerated by the introduction of a high-resolution three-dimensional view (3D). 3D techniques have been improved in comparison to the first generation of 3D vision system introduced in the 90s and can even replace the classic bi-dimensional view [7]. 3rd-generation three-dimensional view was introduced about 10 years ago but few experiences were reported in literature probably due to some limit of this technique. The use of a quite heavy helmet with a head mounted display caused surgeon fatigue [8,9]. 4th-generation three-dimensional system uses more ergonomic glasses and an innovated technology.

Better knowledge of pelvic anatomy, improvements in surgical technique have led to improved oncological results and reduced adverse functional outcomes. Historically, outcomes of continence, erectile function, and oncologic control were the major surgical achievement and were called ‘trifecta’ outcomes. Nowadays, patients with a diagnosis of prostate cancer are younger, healthier and have higher expectations from the advanced minimally invasive surgical technologies. Hence, the ‘pentafecta’ was proposed as a new method of outcomes analysis by adding early complications and positive surgical margins (PSMs) to trifecta [10]. According to these theories, pentafecta has become a new cornerstone in the analysis of urological surgery results.

In this pilot randomized study, we aim to highlight the differences between the standard two-dimensional (2D) with the 4th-generation three-dimensional view (3D) applied to laparoscopic extraperitoneal radical prostatectomy (LERP) in order to assess if the 3D visualization of the operative field could really improve intraoperative and perioperative steps and the pentafecta outcomes.

## Methods

### Patients and technologies

From October 2012 to July 2013, all patients with clinical T1c prostate tumor, belonging to low/intermediate D’Amico risk group, were included in the study. 86 consecutive patients, who met these criteria, underwent LERP. Patients were selected for inclusion via hospital record data. The data were collected in a database and retrospectively analyzed. Fondazione PTV – Policlinico Tor Vergata Ethic Committee approved our clinical study and data collection. In accordance with our institution’s Ethic Committee, informed and signed consent was obtained from each patient prior treatment. A statement of ethical approval covered permission to access patient records and use them for study purposes. All patients constituting the cohort had at least 1 yr follow-up.

Patients were randomized into two groups: the former 2D-LERP (43 pts) operated with the use of 2D-HD Storz® camera with a 10 mm 0° laparoscope; the latter 3D-LERP (43 pts) operated with the use of a 3D-HD Viking® camera with a 10 mm and 0° lens double-channel stereo-laparoscope. The 3D view is achieved with the help of a 3DHD Viking® screen and with the use of polarized glasses. The glasses are filtered; each lens only lets one direction of light pass through the eye, thus maintaining two perspectives of the image and giving a tridimensional vision.

### Procedures: surgery and rehabilitation

All 86 patients were operated by the same surgeon (P.B.) following the same surgical technique of LERP. A 1,5 cm cutaneous incision is made at 1 cm below the inferior margin of the umbilicus and a dilator device is inserted into the preperitoneal space and about 300 ml of air is inflated to develop the space of Retzius (pneumo-Retzius). 4 secondary trocars are then placed under laparoscopic view (2 for each iliac fossa, right and left) in the inverted fan configuration. The endopelvic fascia is incised on each side and bladder neck is dissected and isolated through the “bladder neck sparing” technique. Once the bladder neck is opened close to the prostate, the posterior lip of the bladder neck is lowered to provide access to the interprostatorectal plane. Vasa deferentes and seminal vesicles are isolated and dissected. Then, prostatic pedicles are incised in an anterograde mode with preservation of the neurovascular bundle when it is indicated. Finally, a meticulous preparation of the urethral stump introduces to the vesicourethral anastomosis which is collected in interrupted sutures [11].

The drainage is left in place until leakage is observed, and normally it is removed on second post-operative day. Urinary fistula is defined as prolonged drainage over postoperative day 10. The catheter is normally removed between 7 and 10 days after surgery; in case of urinary fistula a cystography is carried out on 14th and 21st postoperative day and both drainage and catheter are removed at the complete closure of the anastomosis.

Baseline sexual and urinary functions were assessed before LERP with self-administered, validated questionnaires: the International Index of Erectile Function 6 (IIEF-6) and the Incontinence Quality of Life (I-QoL) [12,13].

Pelvic floor muscles exercises were recommended for all patients immediately after catheter removal in order to facilitate continence recovery. After catheter removal, all patients received phosphodiesterase type 5 (PDE5) inhibitors at least three times a week and began penile rehabilitation no later than three weeks after radical surgery by using intracavernous pharmacotherapy (ICP) with Prostaglandin E1 (alprostadil).

### Data collection

Operative and perioperative data. Operative time (OT – from skin to skin closure), anastomosis time (AT – time to complete the anastomosis till the catheter insertion), number of stitches used (NuS), estimated blood loss (EBL) and any intraoperative complication were recorded. Perioperative data include: days of drainage (DD), days of catheterization (DC), hospital stay (HS), pathological staging and complications. Histopathologic staging was performed according to the 2002 TNM system [14].

Pentafecta. The five outcomes included in the analysis of the pentafecta are complications, positive surgical margins (PSMs) and the trifecta outcomes (urinary continence, sexual potency, biochemical recurrence BCR-free survival rates). Pentafecta is achieved if there were no complications, negative surgical margins and if the patient was continent, potent and BCR-free.

### Statistical analysis

Fisher test was used to analyze non-parametric data as appropriate. Student *t*-test was used to analyze parametric data such as patients’ characteristics, intra and remaining perioperative data. Results were considered significant if the p value was ≤ 0.05.

## Results

There were not significant differences between the two groups in terms of age, body mass index (BMI), preoperative PSA level and biopsy Gleason score. Patients’ characteristics are summarized in Table 1.

**Table 1:** **Preoperative data**

**Variable**	**2D**	**3D**
Age, yr, mean	60.1	63.9
BMI, mean	25.2	24.6
Preoperative PSA value (ng/mL)	6.7	6.2
Biopsy Gleason score	6.15	6.15

### Operative and perioperative data

Operative and perioperative data are presented in Table 2. Median OT for 3D-LERP was significantly shorter than that in 2D-LERP (162 versus 241 minutes, p 0.01). Moreover, in the 3D LERP group, median OT for the first 3 cases was significantly longer than the remaining cases due to the initial operator learning curve. Statistically significant differences were also recorded in median AT (24 versus 32 minutes, p 0.03) and median NuS (5.65 versus 6.45, p 0.018). Median EBL did not reach a statistical significance in the two groups with two patients requiring transfusion in the 2D group and 1 patient in the 3D group. No conversion to open surgery was necessary and no complications occurred requiring early re-intervention. Median HS was 7.6 and 5.5 days for the 2D-LERP and 3D-LERP respectively (p = 0.180). Median DD was 5 in the 2D-LERP and 4,5 in the 3D-LERP (p = 0.925). The median CT was of 10.55 days and 10.75 days for the 2D to 3D respectively (p = 0.880).

**Table 2:** **Intraoperative and perioperative data**

**Intraoperative data**			
**Variables**	**2D**	**3D**	**P value**
Mean operative time (min)	241 (150–350)	162 (90–160)	0,01*
Mean anastomosis time (min)	32 (20–45)	24 (15–50)	0,03*
Number anastomosis stitches	6,45	5,65	0,018*
Mean blood loss (ml)	532 (200–2000)	383 (200–600)	0,11
**Perioperative data**			
Days of drainage	4.5	4.85	0.92
Days of catheterization	10.55	10.75	0.88
Hospital stay	7.6	5.5	0.18

*Statistically significant differences.

### Complications

Complications can be considered as a perioperative outcome. We discussed complications apart from the other perioperative data in order to underline their role in the pentafecta.

Modified Clavien grading system was used to classify complications occurring during the surgical procedure or within 90 days after surgery (early complications) [15].

Twenty-three of 86 patients experienced complications. More specifically, perioperative complications were reported in 15 (34.8%) cases in the 2D-LRP and in 8 (18.6%) cases in the 3D-LRP. 2D-LRP and 3D-LRP complications are summarized in Table 3. Minor complications (Clavien grade 1 and 2) represent respectively 80% and 63% for the two groups of all those reported. Major complications (Clavien grade ≥ 3) constituted respectively 20% and 37%. There were no cases of complications graded 4a, 4b and 5 according to the modified Clavien grading system. Data are depicted in Table 3.

**Table 3:** **Complications**

**Modified Clavien grading system**	**2-D**	**3-D**
	**(n = 43)**	**(n = 43)**
**Grade 1**	Penis and scrotum edema	Penis and scrotum edema
	(n = 7)	(n = 5)
	Hematuria (n = 1)	0
	bladder catheter exchange (n = 1)	0
**Grade 2**	transfusions (n = 2)	0
	Epididymitis (n = 1)	
**Grade 3a**	AUR (n = 1)	AUR (n = 1)
	Anastomotic stenosis (n = 2)	Anastomotic stenosis (n = 1)
**Grade 3b**	0	Urinary fistula (n = 1)
**Grade 4a-b/5**	0	0
**Total (%)**	34.8% (15)	18.6% (8)
	Minor 80%	Minor 63%
	Major 20%	Major 37%

### Oncologic outcomes: biochemical recurrence and positive surgical margins

Oncological results are presented in Tables 4 and 5. The distribution of pathological stage and Gleason score was similar in the 2 groups.

**Table 4:** **Histopathologic data**

	**2-D**	**3-D**
	**(n = 43)**	**(n = 43)**
**Pathologic stage**		
**pT2a**	5 (12%)	6 (14%)
**pT2b**	3 (7%)	3 (7%)
**pT2c**	25 (58%)	23 (54%)
**pT3a**	7 (16%)	8 (18%)
**pT3b**	3 (7%)	3 (7%)
**Specimen Gleason score**		
**6 (3 + 3)**	23 (53%)	23 (53%)
**7 (3 + 4)**	8 (18%)	9 (21%)
**7 (4 + 3)**	11 (26%)	9 (21%)
**8 (4 + 4)**	1 (2%)	2 (5%)

**Table 5:** **Oncological results**

	**2-D**	**3-D**
	**(n = 43)**	**(n = 43)**
**Positive surgical margin**	9% (4)	4% (2)
**pT2c**	2/25	1/23
**pT3**	2/10	1/11
**BCR-free rate (%)**		
**3 months follow-up**	93% (40)	95% (41)
**12 months follow-up**	88% (38)	91% (39)

The overall PSM rate was 9% in the 2D-LERP and 4% in the 3D-LERP. When stratified by pathological stage, PSM rate was significantly different in pT2c/pT3 disease between groups (halved in the 3D-LERP compared with the 2D-LRP group).

BCR was defined as two consecutive prostate-specific antigen (PSA) levels of >0.2 ng/ml [16]. The overall BCR-free rate at 3 months was 93% (2D-LERP, 40 patients) and 95% (3D-LERP, 41 patients). Reassessed at 12 months, the overall BCR-free rate was 88% (2D-LERP, 38 patients) and 91% (3D-LERP, 39 patients). Patients with recurrences underwent further salvage therapy with either radiation and/or hormonal treatment. Aligned with the literature data, 25% of the patients with BCR had PSMs.

### Urinary continence

According to the European Association of Urology Guidelines (EAU Guidelines, 2013), urinary incontinence represents a postoperative complication that persists after 1 year in 7.7% of patients who underwent radical prostatectomy [17]. The American Urological Association Guidelines (AUA Guidelines, 2007 updated in 2011) report a rate of postoperative urinary incontinence that ranges between 3% and 74% [18].

Urinary continence was assessed with the self-administered, validated questionnaire Incontinence Quality of Life (I-QoL). The definition of continence was based on a specific question appropriate to reflect the range of incontinence severity: “How many pads per day did you usually use to control urine leakage during the last 4 weeks?”. We considered “dry” patients without any loss of urine (no pads/day) or those who used a safety pad/day.

The overall continence rates did not reach a statistically significant difference although the trend is clearly favorable to the 3D-LERP group (89% and 92% vs 83% and 88% of patients were continent at 3 and 12 month follow-up in the 3D and 2D-LERP group respectively).

I-QoL questionnaire showed a significant quality of life improvement at the first month in the 3D (mean score 90,45) compared to the 2D-LERP group (mean score 81,8) (p = 0.01). These positive results are also confirmed at third (93.3 vs 83.6 - p = 0.01) and twelfth (95.4 vs 88.1 p = 0.03) month follow-up in the 3D compared to the 2D-LERP group respectively. Pre- and postoperative urinary continence data are depicted in Table 6.

**Table 6:** **Continence data**

***Pad system***	**2-D**	**3-D**
	**n = 43**	**n = 43**
**Pre-operative**	100% (43)	100% (43)
**3 months follow-up**	83% (36)	89% (38)
**12 months follow-up**	88% (38)	92% (40)
***IQoL***	**2-D**	**3-D**
	n = 43	n = 43
**3 months follow-up**	83,6	93,3
**12 months follow-up**	88,1	95,4

### Erectile function

It is widely recognized that the preoperative Erectile Function (EF) is an important prognostic factor for erectile dysfunction recovery after radical prostatectomy [19]. Several other factors are predictive for EF recovery after surgery: age, type of surgery, pre- and post-RP libido, adjuvant treatments, comorbidities, urinary continence, availability of a partner and sane mental health. Therefore, it is essential to determine the EF baseline. The International Consultation on Sexual Medicine (ICSM) Committee recommends the use of validated psychometric instruments such as IIEF. In our experience, potency rate has been assessed using the IIEF-6. After surgery, erectile function rehabilitation was recommended for all patients (scheme reported above) in order to preserve the functional smooth muscle tissue of the corpora cavernosa and to avoid the effects of the surgical-related neuroapraxia [20].

Preoperative Potency was defined as the ability to achieve and maintain satisfactory erection for sexual activity or as a score IIEF-6 score ≥ 17 (without pharmacological or mechanical support). Post-operative potency was defined as the ability to achieve and maintain erections firm enough for sexual intercourse in more than 50% of attempts, with or without the use of iPDE5 and with eventual ICP (IIEF-6 score ≥ 17).

Patients were subjected to bilateral or unilateral nerve-sparing surgery (NSS). The overall potency rates were 60% and 67% at 3 months and 67% and 72% at 12 months in the 2D- and 3D-LERP group respectively. Type of surgery, pre-surgical evaluation of erectile function and potency outcomes are summarized in Tables 7 and 8.

**Table 7:** **Potency surgical approach**

**Nerve sparing surgery**	**2-D**	**3-D**
**Monolateral**	42% (18)	37% (16)
**Bilateral**	58% (25)	63% (27)

**Table 8:** **Potency data**

**Erectile Function IIEF 6 ≥ 17**	**2-D**	**3-D**
	**n = 43**	**n = 43**
**Pre-operatory**	86% (37)	88% (38)
**3 months follow-up**	60% (26)	67% (29)
**12 months follow-up**	67% (29)	72% (31)

### Pentafecta outcomes

The overall pentafecta rate at 3 months was 47.4% and 49.6% in the 2D- and 3D-LERP group respectively. The pentafecta rate at 12 months was 62.7% and 67% for each group respectively.

The most common reasons for not achieving the pentafecta were erectile dysfunction (33% and 28% respectively in 2D-LERP and 3D-LERP; trifecta not achieved) and complication rate (34.8% and 18.6%, 2D vs 3D). Furthermore, urinary incontinence (12% and 8%, 2D vs 3D), BCR (12% and 9%, 2D vs 3D) and positive surgical margins (9% and 4%, 2D vs 3D). Results are shown in Table 9.

**Table 9:** **Variables comprising the pentafecta success rates at 12 mo**

**Variable**	**2D LERP**		**3D LERP**	
	**Patients**	**%**	**Patients**	**%**
Complication	28/43	65	35/43	81
PSM	39/43	91	41/43	96
BCR-free rate	38/43	88	39/43	91
Continence	38/43	88	40/43	92
Potency	29/43	67	31/43	72
**Pentafecta**	27/43	62,7	29/43	67

## Discussion

In the past decade, a dramatic shift towards lower-stage tumors has become evident. Currently, men are younger at the time of diagnosis and more interested in less invasive surgical approaches (eg. Laparoscopy, robotics) than they are for the traditional approach [21]. At the same time and more importantly, normal continence and preserving sexual function are fundamental but not the only primary goals of radical prostatectomy. Patients want to know if the treatment option will render them cancer free with a minimum of complications and the shortest possible convalescence time while preserving continence and potency [10].

These observations highlight two main topics: on one hand, the possibility of considering a minimally invasive surgical approach with its innovative technical characteristics every time that it is possible and, on the other hand, the necessity of adopting a more comprehensive method of reporting peri- and post-operative outcomes.

By adopting the laparoscopic technique with adherence to established oncological principles, the aim is to duplicate the open surgical method in its entirety. LRP has slowly risen in popularity and has become, in some centers, the surgical approach of choice for the treatment of localized prostate cancer for its advantages. Lower blood loss and transfusion rate associated with the laparoscopic approach together with shorter hospital stay, reduced catheterization time, better pain control and faster return to everyday activities seem to be the most encouraging improvements obtained [22].

Unfortunately, classic laparoscopic surgery is limited by a two-dimensional vision that does not allow perception of the operative field as in open surgery. The lack of depth perception has repercussions both on the learning curve, which still constitutes a major obstacle to the development of laparoscopy [23], and in the possibility for the surgeon to maneuver the instruments with an accuracy comparable to that which would occur in the same “open” operation. Even if the experienced surgeon is able over time to regain some vision of depth, this will never be optimal [24].

For this reason and through the increasing popularity of laparoscopy, a three-dimensional display system was introduced in the early 90s, with the expectation that this technique could make laparoscopic interventions safer and faster^23^. Up to now, just a few studies on three-dimensional laparoscopy have been written without any definite conclusion about its utility. Some articles describe better results with 3D laparoscopic technique than with the 2D system both in surgical training exercises and in different surgical procedures. Exercises like linear cutting and suturing, curved cutting and suturing, tubular suturing and dorsal vein complex suturing simulation have been performed and it has been suggested that the new-generation 3D system could be helpful in laparoscopy [25-27].

In the 90s, comparative studies were organized to evaluate the improvement and superiority of vision between traditional 2D and 3D system (3rd generation) in terms of dissection of the kidney, securing of the renal vessels and laparoscopic suturing, but the Authors found no differences between the two vision systems, either with respect to the accuracy and speed of surgical execution, nor as regards to the learning curve [28-32]. Gynecologists and general surgeons have described similar studies in the field of 3D laparoscopic surgery with discordant conclusions [33-35].

Robotic surgery had a great benefit from the three-dimensional view. The advent of Da Vinci® has further helped to confine laparoscopic prostatectomy to a special niche. Shorter learning curve, three-dimensional view as well as the ease of movement offered by the operating arms, makes robot-assisted laparoscopic prostatectomy (RALP) more reproducible despite the higher costs. This way, Robertson et al. recently underlined that RALP is easier to learn and is now the surgical treatment of choice in most centers of excellence in the United States [22].

This is the first study reported in urologic literature that aims to establish, after twenty years since the first 3D model was introduced, the utility of the 4th generation 3D vision system during LERP in terms of feasibility and potential advantages over the 2D vision system regarding operative and perioperative data and the pentafecta outcomes. Only one work reported by Good et al. in 2013 analyzed the pentafecta learning curve for laparoscopic radical prostatectomy [36].

Transition from the 2D to 3D vision system, requires an initial period of adaptation. This is demonstrated by longer operative time and the incidence of post-operative urinary fistula that occurred at the very beginning of our experience using the 3D vision system. This short learning curve is related to a new perception of the depth of the operative field that requires a different spatial assessment of instrument positioning rather than an initial difficulty in recognizing anatomical landmarks avoiding possible complications. Once adaptation to 3D view is reached, a more realistic visualization of the surgical field allows greater speed and precision in the movement of the surgical instrument. This translates in a better preparation of the bladder neck and the urethral stamp reducing anastomosis time. Although not resulting in a statistically significant difference, the easy identification of small vessels using the 3D vision may reduce blood loss.

Despite the necessary adaptation from 2D to 3D vision by an expert laparoscopist, on the other hand, the 3D vision may offer significant advantages in teaching laparoscopic skills to inexperienced individuals [37].

Meticulous handling and tissue dissection obtained with the auxilium of the 3D view have allowed earlier continence recovery. This could be mainly related to less trauma and greater sphincter- structures saving [38] as demonstrated by a better I-QoL and a decreased number of pads per day in the 3D LERP Group. One of the operating steps that gets more advantages from the 3D view is the dissection of seminal vesicles, vas deferens and prostatic pedicles. Dissection of these delicate structures makes 3D vision very effective. Basically, these operating stages and their higher accuracy might affect a possible earlier and better recovery of erectile function.

These encouraging results obtained with the 3D vision system were associated with a number of positive surgical margins and post-operative complications comparable in both groups demonstrating a good oncological and functional efficacy. From our point of view, some problems related to the prolonged use of 3D vision such as headaches, fatigue and nausea, already reported in previous studies, have still remained unresolved, but it is not an important limitation to its use [39,40].

Statistically significant differences were recorded for all intraoperative steps and data suggest a trend of improvement in intraoperative blood loss, postoperative recovery of continence and potency with the respect of the oncological safety for the 4th generation 3D-HD vision system of the 3D-LERP over standard bi-dimensional view in 2D-LERP.

One of the advantages of this study is that the comparison between the 2D and 3D surgical procedures was performed by a single surgeon making it more reliable and avoiding possible bias. Despite this fact, the extensive experience of the surgeon may have influenced the results and complication rates of our study and, as a result, the outcomes cannot be generalized.

However, this study has several limitations. First of all, being a pilot study, with a small number of procedures and a relatively short follow-up, it does not allow the definition of the definitive role of this technique. Data analysis was a retrospective. Some data may not reach a statistical significance between groups because the study was not powered to identify these differences; nevertheless, a trend of improvement in surgical and functional outcomes has been shown. Furthermore, we included both bilateral and monolateral NSS procedures. Another limitation is that we did not use the Expanded Prostate Cancer Index Composite (EPIC) questionnaire to better assess urinary symptoms. Finally, the study is limited by the short follow-up, which can affect BCR-free and functional outcomes.

Nowadays, all the laparoscopic prostatectomies in our Department are performed with the auxilium of 3D video system. If these preliminary data will be confirmed by larger follow-up of a greater number of patients, the 4th generation 3D laparoscopy may play an important role in the treatment of prostate cancer.

## Conclusions

This preliminary study has shown that 4th generation 3D-HD vision system provides advantages over standard bi-dimensional view with regard to intraoperative steps. Our data suggest a trend of improvement in intraoperative blood loss and early postoperative recovery of continence along with the respect of the oncological safety. Pentafecta has been reached with a higher score for the 3D-HD LERP.

Given the large number of men diagnosed with prostate cancer and the exponential growth of the medical costs on a global level, it would be better that treatment options are not only effective but also less expensive. In this context, the 3D laparoscopy may be an intermediate step between the standard 2D laparoscopy and robot-assisted laparoscopy, allowing the combination of the low cost of the first with the 3D technology of the second. Further studies are necessary to better comprehend the role of 3D-LERP in modern urology.

## Abbreviations

LRP: Laparoscopic radical prostatectomy
LERP: Laparoscopic extraperitoneal radical prostatectomy
RRP: Radical retropubic prostatectomy
RALP: Robot-assisted laparoscopic prostatectomy
3D HD: High-definition three dimensional view
PSMs: Positive surgical margins
IIEF 6: International index of erectile function 6
IQoL: Incontinence quality of life
PDE5: Phosphodiesterase type 5
ICP: Intracavernous pharmacotherapy
OT: Operative time
AT: Anastomosis time
NuS: Number of stitches
EBL: Estimated blood loss
DD: Days of drainage
DC: Days of catheterization
HS: Hospital stay
BCR: Biochemical recurrence
BMI: Body mass index
EAU: European association of urology
AUA: American urological association
ICSM: International consultation on sexual medicine
NSS: Nerve-sparing surgery
EPIC: Expanded prostate cancer index composite
